# A Microfabricated Bandpass Filter with Coarse-Tuning and Fine-Tuning Ability Based on IPD Process and PCB Artwork

**DOI:** 10.3390/mi13010123

**Published:** 2022-01-13

**Authors:** Junzhe Shen, Tian Qiang, Minjia Gao, Yangchuan Ma, Junge Liang, Yanfeng Jiang

**Affiliations:** Department of Electronic Engineering, School of Internet of Things Engineering, Jiangnan University, Wuxi 214122, China; 1038190121@stu.jiangnan.edu.cn (J.S.); 6201924078@stu.jiangnan.edu.cn (M.G.); 6201924123@stu.jiangnan.edu.cn (Y.M.); jgliang@jiangnan.edu.cn (J.L.); yanfeng_jiang@yahoo.com (Y.J.)

**Keywords:** bandpass filter, asymmetrical spiral inductor, interleaved array capacitor, coarse-tuning, fine-tuning

## Abstract

In this paper, a bandpass filter (BPF) was developed utilizing GaAs-based integrated passive device technology which comprises an asymmetrical spiral inductor and an interleaved array capacitor, possessing two tuning modes: coarse-tuning and fine-tuning. By altering the number of layers and radius of the GaAs substrate metal spheres, capacitance variation from 0.071 to 0.106 pF for coarse-tuning, and of 0.0015 pF for fine-tuning, can be achieved. Five air bridges were employed in the asymmetrical spiral inductor to save space, contributing to a compact chip area of 0.015λ_0_ × 0.018λ_0_. The BPF chip was installed on the printed circuit board artwork with Au bonding wire and attached to a die sink. Measured results demonstrate an insertion loss of 0.38 dB and a return loss of 21.5 dB at the center frequency of 2.147 GHz. Furthermore, under coarse-tuning mode, variation in the center frequency from 1.956 to 2.147 GHz and transmission zero frequency from 4.721 to 5.225 GHz can be achieved. Under fine-tuning mode, the minimum tuning value and the average tuning value of the proposed BPF can be accurate to 1.0 MHz and 4.7 MHz for the center frequency and 1.0 MHz and 12.8 MHz for the transmission zero frequency, respectively.

## 1. Introduction

Bandpass filters (BPFs), as a crucial part of radiofrequency (RF)/microwave systems, are widely applied in wireless communication with multiple functions. However, BPFs face ongoing challenges in terms of achieving better efficacy, including higher performance, more compact size, and more flexible tuning [1,2,3,4]. Among these demands, the significance of the first two aspects has been fully discussed in numerous papers, whereas excellent tunability, which can facilitate BPFs in frequency selection under diverse operation environments, is playing an increasingly vital role. Therefore, the design of BPFs with a high performance, small size, and excellent tunability is the target of our research work.

There have been various schemes reported aiming to develop such a BPF with excellent tunability. Tuning of the center frequency and bandwidth has been widely researched in recent studies [5,6,7]. In [5,6], substrate-integrated waveguide (SIW) cavities with E-shaped slots and a cantilever-based RF micro-electromechanical system (MEMS) switched capacitor were applied, through which center frequency tuning was successfully realized. In [7], a compact quad-band utilizing multi-stub-loaded resonators (SLRs) was proposed. With four controllable modes and multiple coupling paths between different stubs, passband frequency tuning and bandwidth tuning can be accomplished. Nevertheless, there have been relatively few efforts devoted to the tunability of both the center frequency and transmission zero frequency. Moreover, the majority of studies to date tended to focus on wide-tuning [8,9,10,11,12,13,14], whereas few studies have contributed to fine-tuning. In [8,9], p-i-n diodes as tuning elements were used in a switched tunable diplexer and a cross-shaped resonator with open stubs, separately. In [10,11], a BPF based on even–odd mode theory and microstrip technology was developed, in which varactor diodes were applied for a wide tuning range. Additionally, some other methods have also been utilized in the field of wide-tuning, such as ferroelectric capacitors with tunable CSRR scatterers [12], a dual-band, dual-mode SIW balanced BPF tuned by vias and slot perturbations [13], and tunable coaxial cavity resonator-based filters by altering the volume of Galinstan [14]. Nevertheless, these tuning schemes possess some inevitable shortcomings, including relatively high insertion loss (IL) [8,9,11,12,13], poor out-of-band rejection and upper stopband [10], high cost and cumbersome process [14], and a large interval between two tuning values, in particular [8,9,10,11,12,13,14]. To further make the tuning value more accurate, a novel microfluidic tunable differential BPF with a precisely tunable center frequency has been proposed [15]. By inserting microfluidic channels which are padded with a high-dielectric constant fluid, frequency tuning states can be appropriately controlled. However, their relatively large circuit area and complex fine-tuning modes may confine their applications in modern integrated circuits. Recently, some researchers have been dedicated to BPF implementation utilizing the integrated passive device (IPD) process. IPDs are a promising fabrication technology that strongly promote the minimization of device size and effectively optimize the device performance. In [16,17], a GaAs-based BPF with air bridge structures using IPD technology was proposed with a miniaturized chip size and high-performance characteristics. Moreover, tunability of both the center frequency and transmission zero frequency can be realized by altering the number of turns of the capacitor and the line space of the inductor, respectively. Nevertheless, both of them are still confronted with the dilemma of fine-tuning realization.

In this work, we developed a tunable BPF using GaAs-based IPD technology with two tuning modes—coarse-tuning and fine-tuning. The proposed BPF is the combination of an asymmetrical spiral inductor and an interleaved array capacitor. To realize the two tuning modes, we chose to alter the capacitance to control the coarse-tuning and fine-tuning of the center frequency and transmission zero frequency. Multiple methods for altering the capacitance were compared and tested, and, ultimately, two of the most effective tuning modes were found and built. Additionally, an equivalent circuit was constructed for the BPF model, and the processing technology was briefly discussed and applied to obtain a higher yield. Furthermore, the fabricated BPF chip was assembled on a sub-board PCB through Au wire bonding technology for practical measurement and performance evaluation. Finally, compared with other BPFs, our BPF demonstrates its superiority in tunability including coarse-tunability and fine-tunability.

## 2. Design and Analysis

### 2.1. BPF Design and Processing

**Figure 1:**
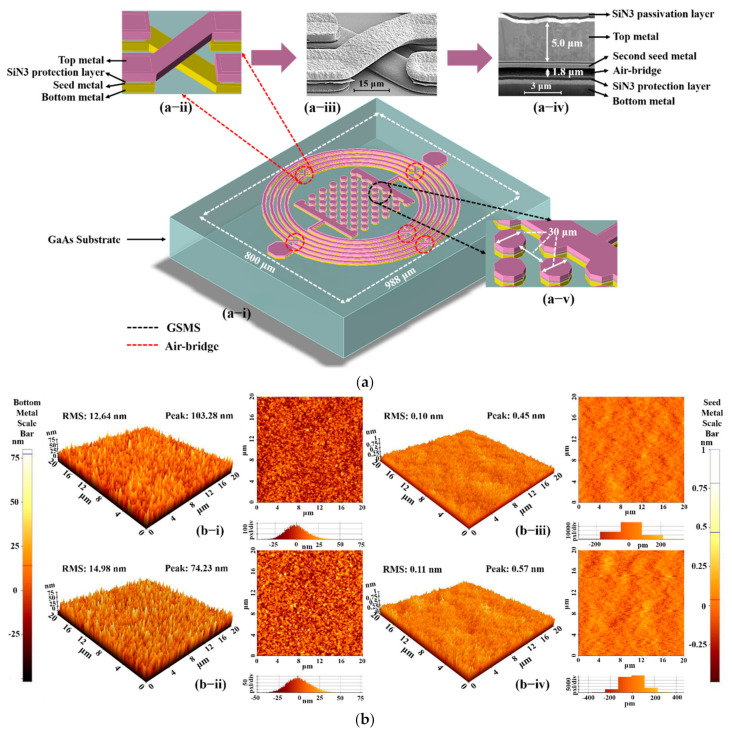
(**a**) Detailed information of proposed BPF: (**a-i**) 3D structure of the proposed BPF, (**a-ii**) magnified view of air bridge, (**a-iii**) SEM image of air bridge section, (**a-iv**) FIB image of air bridge section, (**a-v**) enlarged view of GSMS. (**b**) AFM images of fabricated BPF: (**b-i**) original bottom metal without Ar sputter etching process, (**b-ii**) current bottom metal with Ar sputter etching process, (**b-iii**) original seed metal without Ar sputter etching process, (**b-iv**) current seed metal with Ar sputter etching process.

As demonstrated in Figure 1a, the proposed BPF is composed of an asymmetrical spiral inductor and an interleaved array capacitor, which maintains a compact area of 800 × 988 μm. The inductor was fabricated using 5-turn intertwined meander lines with a line width of 15 μm, line space of 15 μm, inner diameter of 275 μm, and outer diameter of 425 μm. Five air bridges were applied to the inductor to obtain a higher Q-factor and ensure connection. An enlarged 3D structure of the air bridge is provided in Figure 1a-ii, where the top metal layer is marked in purple, while the bottom metal layer is marked in yellow, with the SiN3 protection layer and the seed metal layer in the middle. In addition, to further observe the actual structure of the proposed BPF, an image of an enlarged air bridge section taken by a scanning electron microscope (SEM) is shown in Figure 1a-iii, and a cross-sectional image by focused ion beam (FIB) with an air bridge gap of 1.8 μm and a top metal thickness of 5 μm is shown in Figure 1a-iv. In this work, a 0.2 μm SiN3 protection layer was designed to protect the bottom metal surface and sidewall from air oxidation, and a 0.3 μm SiN3 passivation layer was developed to protect the whole device from air oxidation and physical scratch. Moreover, the second Ti/Au seed metal was utilized for subsequent electroplating of the top metal. After electroplating, the excess seed metal was etched by the reactive ion etching (RIE) method to prevent short circuits. Nevertheless, due to the limited opening depth of FIB, the first seed metal is not displayed in the image. Moreover, the interleaved array capacitor contains 49 GaAs substrate metal spheres (GSMS); a magnified view is provided in Figure 1a-v, with a 15 μm radius on 30 μm center intervals.

During processing, insufficient adhesion between the bottom metal layer and the top metal layer triggers the peel-off issue of the top metal, which will reduce the fabrication yield. Therefore, appropriate processing technology was utilized to enhance the adhesion in the effective area. We provide two reasonable assumptions: (1) there is insufficient adhesion between the bottom metal and the second seed metal; (2) there is insufficient adhesion between the second seed metal and the top metal. Figure 1b presents the AFM images of distinct metal layers, where the Ar sputter etching process was tested to cope with the peel-off issue. It was found that the root mean square (RMS) of the bottom metal increased from 12.64 to 14.98 nm, whereas the increase in the seed metal RMS was not significant. Additionally, a higher RMS value reflects a metal surface with a higher roughness; therefore, adhesion can be enhanced [18], and the peel-off issue can be ameliorated using the Ar sputter etching process on the bottom metal layer. Meanwhile, a lower metal peak value was achieved, which effectively reduces the risk of short circuits in the following metal process.

### 2.2. Tuning, Modeling, and Simulation

**Figure 2:**
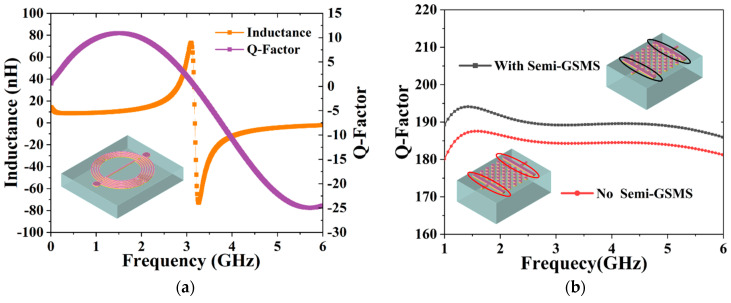
(**a**) Simulated inductance and Q-factor of the asymmetrical spiral inductor. (**b**) Simulated Q-factor of the capacitor with semi-GSMS and without semi-GSMS.

The asymmetrical spiral inductor is modeled and simulated in Figure 2a, where the inductance and Q-factor can be determined using 3D EM simulation by

(1) $$Inductance=\frac{imagZ_{11}}{2\times \pi \times freq}$$

(2) $$Q-factor_{L}=\frac{imagZ_{11}}{realZ_{11}}$$

It can be seen in the figure that the Q-factor of the inductor peaks at 1.52 GHz, where the related inductance is 10.60 nH. Nevertheless, to maintain a compact chip size, a trade-off should be established among the inductance, the Q-factor, and the self-resonant frequency. In this work, the center frequency of 2.147 GHz was employed, which can be coarse-tuned and fine-tuned by altering the capacitor. The interleaved array capacitor consists of two rectangular parallel plates and a staggered GSMS array, which is composed of 49 GSMS. Additionally, 6 semi-GSMS were designed and attached to each plate to improve the Q-factor of the capacitor. Figure 2b presents the simulated Q-factor of two diverse capacitors with and without semi-GSMS. It was found that the Q-factor of the capacitor with semi-GSMS is higher than that of the capacitor without semi-GSMS at the operating frequency of 2.147 GHz, contributing to the improvement in the Q-factor of the resonant circuit and enhancement of the insertion loss performance [19,20]. This could be attributed to electromagnetic effects between semi-GSMS and GSMS. The calculation formula of the capacitor is given as [21]

(3) $$Q-factor_{C}=\frac{imagY_{11}}{realY_{11}}$$

**Figure 3:**
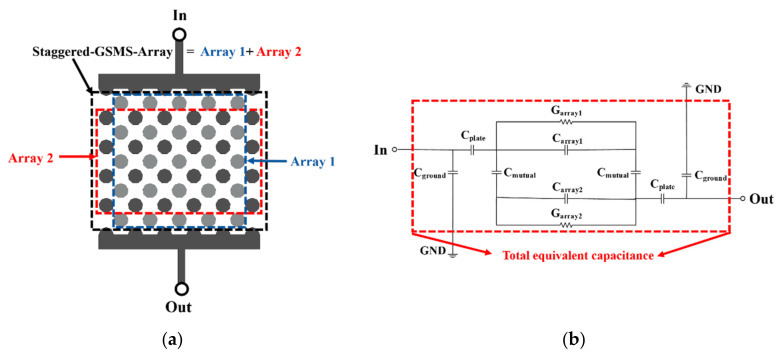
(**a**) Top view of the interleaved array capacitor and (**b**) its equivalent circuit model.

Figure 3a demonstrates a 2D top view of the interleaved array capacitor. The staggered GSMS array is divided into 2 arrays for equivalent circuit analysis, which are distinguished by two different colors. The equivalent circuit model of the proposed capacitor is illustrated in Figure 3b. Here, *C_plate_* stands for the capacitance between the plates and arrays, *C_array_*_1,2_ represents the capacitance of the staggered GSMS array with the leakage conductance of *G_array_*_1,2_, *C_ground_* denotes the capacitance effect caused by the direct magnetic flux from the signal electrode to the grounding electrode of the interleaved array capacitor, and *C_mutual_* is the inductive capacitance formed by the two arrays. The total equivalent capacitance is the equivalent capacitance formed by the interaction of the above four capacitances.

**Figure 4:**
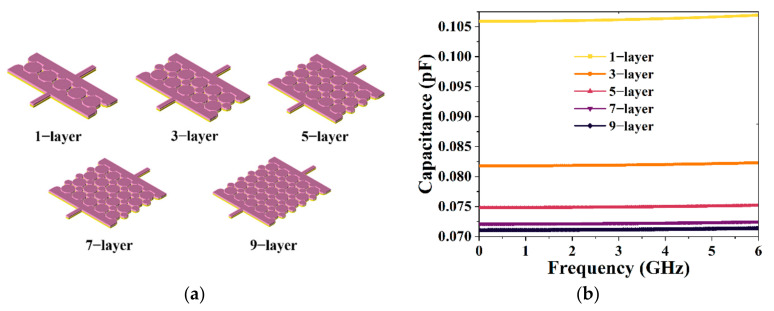
(**a**) Three-dimensional structure of the proposed capacitor with 1-layer, 3-layer, 5-layer, 7-layer, and 9-layer GSMS, as well as their corresponding (**b**) capacitance performances.

**Figure 5:**
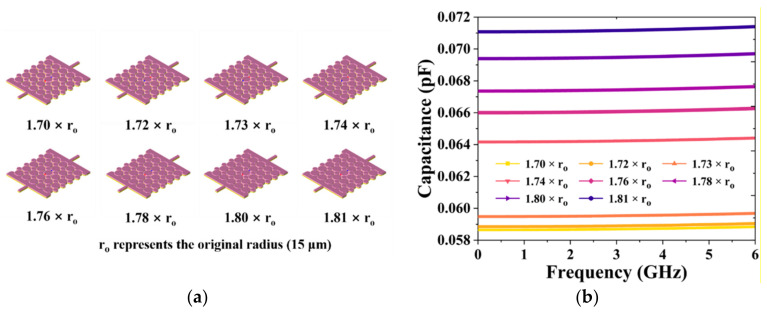
(**a**) Three-dimensional structure of the proposed capacitor with 1.70-, 1.72-, 1.73-, 1.74-, 1.76-, 1.78-, 1.80-, and 1.81-radius GSMS, as well as their corresponding (**b**) capacitance performances.

In order to obtain an excellent tunable performance for our proposed BPF, including excellent coarse-tuning and fine-tuning performance, the capacitor was designed to meet the demand for small- and large-range variation. The center frequency and transmission zero frequency of the BPF are controlled by adjusting the capacitance. Therefore, we tested the effects of a series of diverse arrangement modes, center intervals, radii, and numbers of GSMS on the capacitance parameters and finally obtained the two most effective tuning modes, corresponding to coarse-tuning and fine-tuning. For the large-range variation in the capacitance, we altered the number of layers of the staggered GSMS array and expanded the GSMS radius of odd layers to 1.81 times that of the original. For the small-range variation in the capacitance, we expanded the GSMS radius of the odd layers to 1.70~1.81 times that of the original while keeping the same number of layers, as shown in Figure 4 and Figure 5. Figure 4a demonstrates the 3D structures of the capacitor with GSMS of various layers of 1, 3, 5, 7, and 9, corresponding to the numbers of GSMS of 5, 16, 27, 38, and 49, respectively. The related capacitance can be calculated by

(4) $$Capacitance=\frac{imagY_{11}}{2\times \pi \times freq}$$

Therefore, the capacitance performance was simulated as illustrated in Figure 4b. It can be seen in Figure 4 that variation from 0.071 to 0.106 pF for the capacitance can be achieved while changing the number of layers of the staggered GSMS array. As shown in the figure, the capacitance decreases more slowly when the number of layers is increased from 1 to 9, which can be attributed to the difference in 1/d (d denotes the distance between the parallel plates) between the two adjustment decreases. Meanwhile, Figure 5a provides a 3D view of the proposed capacitor with 9-layer GSMS of diverse radii of 1.70, 1.72, 1.73, 1.74, 1.76, 1.78, 1.80, and 1.81 times the original, and the corresponding capacitance performance is exhibited in Figure 5b. Note that, for the small-range variation, a total of 8 * 5 = 40 groups of capacitances were simulated, while Figure 5 depicts the capacitance performance based on 9-layer GSMS (1 * 8 = 8 groups in total). Moreover, an average variation of 0.0015 pF can be achieved for the sake of small-range variation. These results prove that our proposed capacitor possesses excellent wide-range variation capacity for BPF coarse-tuning and accurate regulation ability for BPF fine-tuning.

### 2.3. BPF Equivalent Circuit

**Figure 6:**
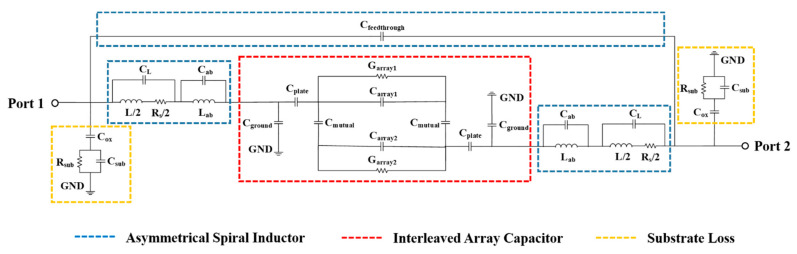
Equivalent circuit of the proposed BPF.

Figure 6 demonstrates the equivalent circuit model of the proposed BPF. To simplify the analysis, the equivalent circuit model is divided into three parts: inductance, capacitance, and substrate loss, which are shown with distinct colors. The equivalent circuit analysis of the interleaved array capacitor is fully discussed above, and thus we focus on the equivalent circuit of the inductance and substrate loss in this section. 

For the spiral inductance, it can be approximately equivalent to the circular inductance for calculation. In [22], it was calculated by the following formula

(5) $$L=6.28n^{2}D_{av}\left(20\rho^{2}-100ln\rho +90.02\right)\;\left(nH\right)$$

where *n* stands for the number of turns, *ρ* represents the fill ratio, and *D_av_* denotes the average diameter of the inductor, which can be calculated using

(6) $$\rho =\frac{D_{o}-D_{i}}{D_{o}+D_{i}}$$

(7) $$D_{av}=\frac{1}{2}\left(D_{o}+D_{i}\right)$$

where *D_o_* is the outside diameter of the inductor, and *D_i_* is the inside diameter of the inductor. In the figure, *L_ab_* and *C_ab_* stand for the inductance and capacitance of air bridges, respectively. As for the substrate loss, the proposed BPF generates various parasitic effects at high frequencies owing to its multilayer structure [23]. The parasitic capacitance formed between the planar pattern and the lossy substrate is represented by *C_ox_* and was determined by [17].

(8) $$C_{ox}=\frac{1}{2}lW\frac{\varepsilon_{SiN3}}{t_{SiN3}}$$

where *l* represents the total circle length, *W* stands for the conductor width, and *ε_SiN3_* and *t_SiN3_* denote the permittivity and thickness of the SiN3 layer, respectively. Meanwhile, the resistance and inductance of the GaAs substrate [24] are also taken into account, which take the form of *R_sub_* and *C_sub_* and can be derived as [25,26]

(9) $$R_{sub}=\frac{2}{lWG_{0}}$$

(10) $$C_{sub}=\frac{1}{2}lWC_{0}$$

where *C*_0_ and *G*_0_ are the capacitance and conductance per unit area of the GaAs substrate, respectively. Therefore, after analyzing the equivalent circuit model of the capacitance, inductance, and substrate loss, the resonant frequency of the BPF can be calculated accordingly. Based on a low-order resonator, the BPF can be simplified as a combination of an inductance and a capacitance, and thus the resonant frequency (center frequency) *f_0_* can be obtained by the following formula:

(11) $$f_{0}=\frac{1}{2\pi \sqrt{L_{T}C_{T}}}$$

where *L_T_* and *C_T_* denote the total inductance and capacitance, respectively. Moreover, the frequency of transmission zero can be calculated using [27]

(12) $$\omega_{0}=\omega_{mid}\frac{\Omega_{c}\times FBW+\sqrt{{\left(\Omega_{c}\times FBW\right)}^{2}+4}}{2}$$

where *ω_mid_* and Ω*_c_* represent the mid-band frequency and passband cut-off frequency of the higher-frequency area, respectively, and *FBW* indicates the fractional bandwidth, which can be calculated by

(13) $$FBW=\frac{\omega_{2}-\omega_{1}}{\omega_{0}}$$

(14) $$\omega_{0}=\sqrt{\left(\omega_{1}\omega_{2}\right)}$$

where *ω*_0_ is the operation frequency, and *ω*_2_ and *ω*_1_ are the passband edge angular frequencies. Therefore, based on the design of the above BPF, the capacitance *C_T_* can be altered flexibly, in order to realize tunable parameters and the desired performance of the BPF.

## 3. Results and Discussion

### 3.1. Measurement Preparation and Setup

**Figure 7:**
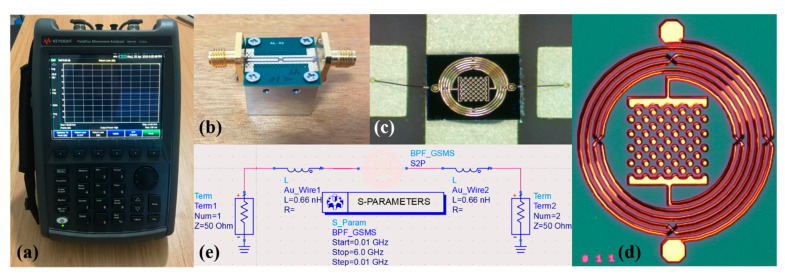
(**a**) Image of the measuring instrument VNA, (**b**) image of the PCB connected to an iron cube with a mounted BPF chip, (**c**) enlarged view of the BPF chip with bonding wire, (**d**) microscope image of the proposed BPF, and (**e**) re-simulation schematic with bonding wire.

The proposed BPF was designed and simulated using Advanced Design System (ADS) 2020 and then manufactured and assembled for practical measurement and data recording. The filter was designed on a GaAs substrate with a dielectric constant of 12.85, loss tangent of 0.006, and thickness of 200 μm. The performance and parameters of the BPF were measured using an Agilent 8510C vector network analyzer (VNA). Figure 7a provides a picture of the measuring instrument VNA, and an image of the BPF assembly is shown in Figure 7b, where the wire-bonded BPF is installed on a sub-board PCB and then attached on a die sink composed of an iron cube. As an additional and expanded grounding, the iron cube has an effect on reducing loss. Furthermore, in order to connect the BPF chip with the PCB, approximately 400 μm-length Au bonding wire with excellent transmission performance was applied in this work, which is shown in Figure 7c. Meanwhile, a microscope image of our fabricated BPF is displayed in Figure 7d. Then, to further make the simulation accord with the actual condition, Figure 7e shows the ADS schematic diagram simulation considering the bonding wire, which is equivalent to a 0.66 nH inductor, and a 50 Ω impedance matching transmission line which is connected to an input/output port. In the measurement, the 200 µm bonding wire is equivalent to a 0.33 nH inductor. Therefore, a 0.66 nH inductor corresponding to approximately 400 µm Au bonding wire was implemented in the re-simulation. Since the PCB was applied to carry chips and actually be measured, the performance of the PCB is also critical.

### 3.2. PCB Design, Simulation, and Measurement

**Figure 8:**
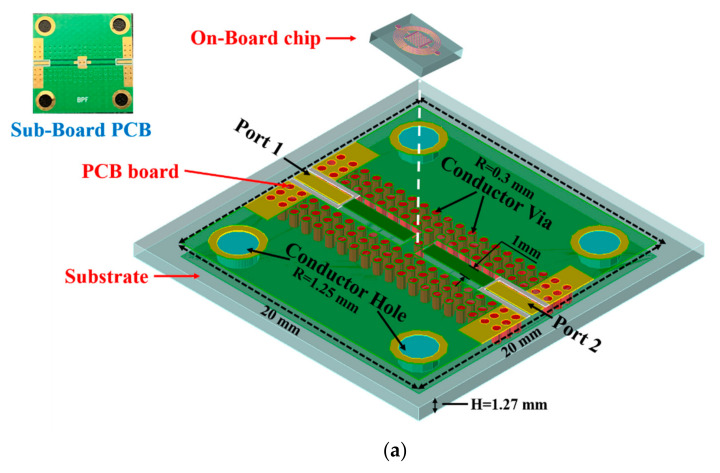

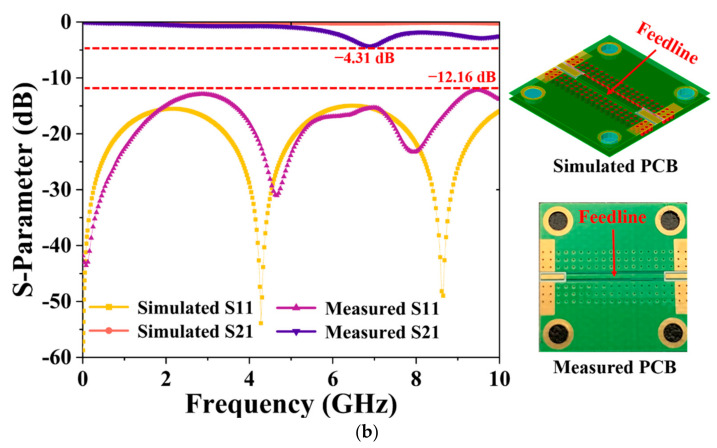
(**a**) Three-dimensional structure of PCB artwork with on-board chip. (**b**) Simulated and measured S-parameter of the PCB with connected feedline.

A 3D view of our proposed sub-board PCB is presented in Figure 8a, and an actual PCB image is shown in the inset image. The substrate used in the PCB artwork is RT/Duriod 5870 with a dielectric constant of 3.5, loss tangent of 0.0018, thickness of 1.27 mm, and total dimensions of 20 × 20 mm. A total of 120 conductor vias with a radius of 0.3 mm and a center interval of 1 mm are distributed on both sides of the feedlines for the sake of microwave constraint and loss reduction. Additionally, four conductor holes with a radius of 1.25 mm were designed on the four corners of the PCB board for the screw installation and to further fix the device under test. The BPF chip, installed on the PCB board with conductive paste, is connected to the feedlines with Au bonding wire. Additionally, a conductor hole is grounded under the BPF chip to further ensure the ground connection. To prove the proposed PCB has minuscule signal loss when transmitting microwave signals, we connected two feedlines to form a pathway, as shown in Figure 8b. The simulation and measurement results are illustrated in Figure 8b. It is evident from the graph that in the frequency range from 45 MHz to 10 GHz, both the simulated and measured return losses are less than −12.16 dB, and the insertion loss is exceedingly close to 0 dB. There is a fluctuation in the insertion loss at 6.86 GHz, which can be attributed to calibration and welding errors. The results successfully prove that our designed PCB possesses excellent transmission performance; thus, it can be applied to carry the BPF chip.

### 3.3. Measurement Preparation and Setup

**Figure 9:**
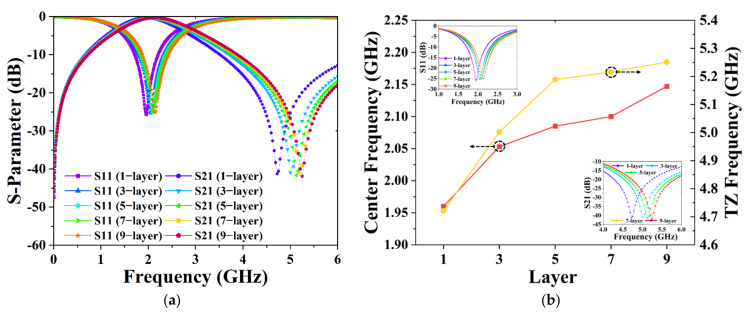
(**a**) BPF performance based on coarse-tuning. (**b**) Results of center frequency and transmission zero frequency based on coarse-tuning, as well as a partial enlarged view of the S-parameter.

**Figure 10:**
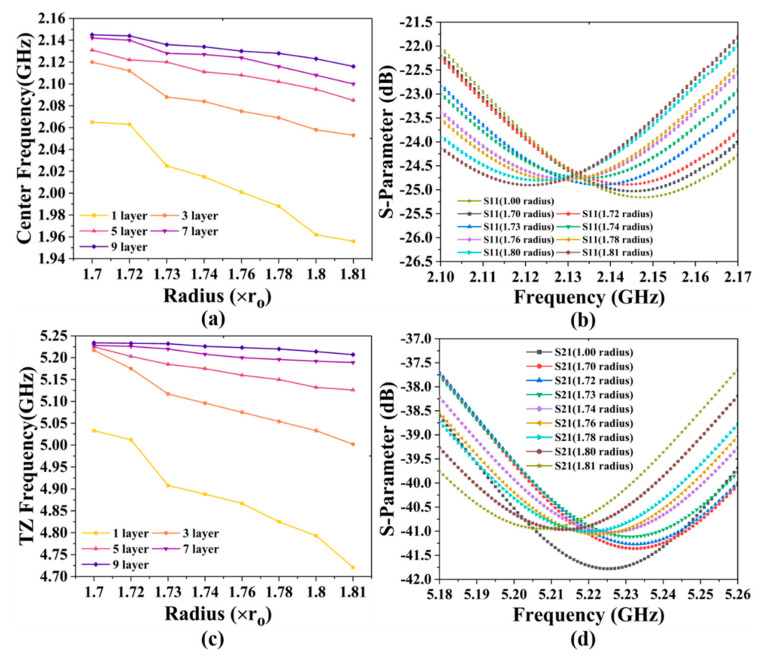
Results of (**a**) center frequency and (**c**) transmission zero frequency based on fine-tuning, as well as specific (**b**) S11-parameter and (**d**) S21-parameter results with 9-layer GSMS.

To prove the feasibility of coarse-tuning and fine-tuning of our proposed BPF, a staggered-array capacitor with GSMS of various layers and radii was designed and simulated to further test and verify the BPF’s performance. In order to realize the function of coarse-tuning, we simulated the capacitor with GSMS of various layers of 1, 3, 5, 7, and 9, corresponding to the numbers of GSMS of 5, 16, 27, 38, and 49, respectively. Based on coarse-tuning, for the same layers of GSMS, we altered the radius to be 1.70, 1.72, 1.73, 1.74, 1.76, 1.78, 1.8, and 1.81 times that of the original GSMS to achieve fine-tuning. As depicted in Figure 9, variation in the center frequency from 1.956 to 2.147 GHz and transmission zero frequency from 4.721 to 5.225 GHz can be achieved, validating the proposed BPF design’s effectiveness in coarse-tuning. Figure 10a shows the center frequency can be tuned more accurately within the coarse-tuning range of 1.956 to 2.147 GHz by changing the radii of GSMS. Lines of different colors correspond to different coarse-tuning states, while different points on lines of the same color represent different fine-tuning states. Therefore, there are 40 different fine-tuning states of the center frequency in the figure, which effectively verifies the practicable fine-tuning of the proposed BPF. 

Furthermore, to quantitatively measure the proposed BPF fine-tuning and coarse-tuning capabilities, here, we present the physical quantity tuning accuracy ratio (TAR), which can be calculated with the following formulas:

(15) $$TAR=\frac{ATV}{R_{c}}\times 100\%$$

where *R_c_* stands for the maximum range of tuning, and *ATV* denotes the average tuning value, which can be determined by

(16) $$ATV=\frac{\sum_{1}^{k}TV_{i}}{k-1}\left(i=1,2,3\ldots ,k\right)$$

where *k* is the number of fine-tuning states, and *TV_i_* is the tuning value in the *i* state. It can be stated that the tuning accuracy ratio decreases as the maximum range of tuning increases or the average tuning value decreases.

Therefore, TAR is an effective index applied to reflect coarse-tuning and fine-tuning abilities, and our proposed BPF was calculated to have a TAR of 2.5% for both the center frequency and transmission zero frequency. Similarly, the transmission zero frequency by fine-tuning within the coarse-tuning range of 4.721 to 5.225 GHz is exhibited in Figure 10c. Specifically, Figure 10b,d are enlarged views of the S11-parameter with 9-layer GSMS and the S21-parameter with 9-layer GSMS by fine-tuning, corresponding to the center frequency and transmission zero frequency, respectively. In addition, it can be calculated from the data that the minimum tuning value of our BPF is 1.0 MHz and 1.0 MHz, and the average tuning value is 4.7 MHz and 12.8 MHz, for the center frequency and transmission zero frequency, respectively.

**Figure 11:**
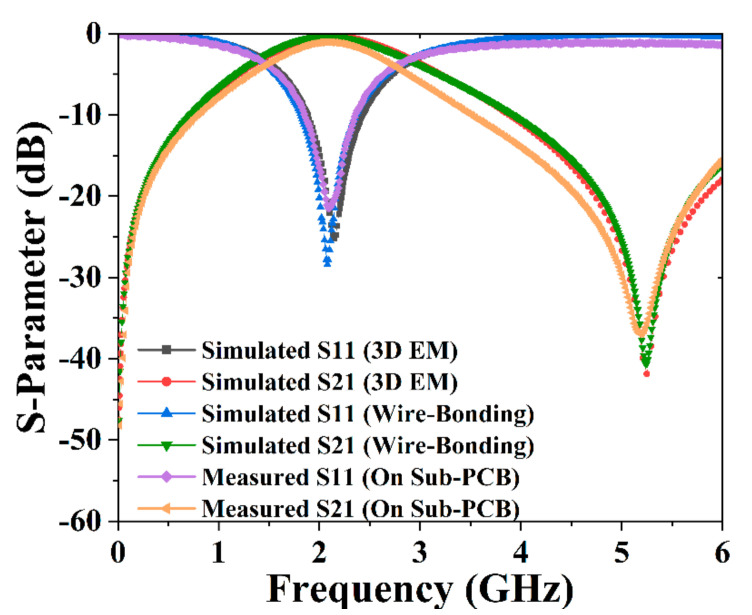
Simulated and measured S-parameter for the proposed BPF.

Figure 11 presents the simulated and measured results of our proposed BPF. The results demonstrate that the center frequencies were 2.147 GHz, 2.080 GHz, and 2.100 GHz based on the 3D EM, schematic simulation considering wire bonding, and sub-board PCB measurement. Meanwhile, the transmission zero frequencies were 5.251 GHz, 5.240 GHz, and 5.188 GHz, respectively. The related return losses of 25.231 dB, 28.401 dB, and 21.496 dB were obtained. Additionally, corresponding insertion losses of 0.320 dB, 0.305 dB, and 0.384 dB could be observed, respectively. Among the above three results, the maximum deviation of the center frequency is less than 0.067 GHz, and that of the transmission zero frequency is less than 0.063 GHz, which proves the accuracy of 3D EM simulation modeling and schematic simulation considering wire bonding. Furthermore, the measured results are in good agreement with the simulated results, validating the low loss of the fabricated PCB artwork and the accuracy of the actual measurement. Moreover, the 3dB passband was 1.406–2.974 GHz for the measured results, with a wide fraction bandwidth (FBW) of 74.67%. The wide FBW contributes to covering various applications around 2 GHz while maintaining a compact dimension. Additionally, the TZ frequency around 5 GHz sacrifices some of the selectivity performance of the proposed BPF, whereas the performance of the out-of-band suppression is successfully enhanced. Moreover, the TZ frequency could be tuned through the variation in the interleaved array capacitor or the introduction of higher-order basic elements, which, reversely, could lead to a relatively large chip size.

Table 1 list the results in comparison with the performance of other reported BPFs in terms of the detailed substrate and process, insertion loss (IL), return loss (RL), chip area, fraction bandwidth (FBW), center frequency controllability (CFC), transmission zero frequency controllability (TZC), tuning accuracy ratio (TAR), minimum tuning value (MTV), and average tuning value (ATV). It can be seen in the table that our designed BPF possesses a relatively low insertion loss, high return loss, compact chip size, and wide fraction bandwidth. Meanwhile, the tunability of both the center frequency and transmission zero frequency is successfully achieved. Moreover, in comparison with other devices, the designed BPF shows a superior performance in tunability. The relatively lower MTV and ATV prove the excellent fine-tuning ability of the proposed BPF; thus, both the center frequency and transmission zero frequency can be tuned accurately. Additionally, the relatively lower TAR proves that the center frequency and transmission zero frequency can be tuned in a wide range of frequency, which validates that the BPF is competitive in tuning applications.

## 4. Conclusions

In this paper, GaAs-based IPD technology was applied to the fabrication of our proposed BPF which is composed of an asymmetrical spiral inductor and an interleaved array capacitor. Coarse-tuning and fine-tuning modes were introduced to the proposed BPF, contributing to the realization of relatively wide-range variation and subtle changes for both the center frequency and transmission zero frequency. In addition, the Ar sputter etching process was applied to handle the peel-off issue, which successfully enhanced the fabrication yield. Moreover, the equivalent circuit with approximate analysis was modeled and calculated. Finally, the BPF chip was mounted on a sub-board PCB artwork which was connected to a die sink of an iron cube. The measurement results are in good agreement with the simulated results, which show an excellent RF performance with a high return loss, low insertion loss, wide FBW, and excellent coarse-tuning and fine-tuning, in particular.

## Figures and Tables

**Table 1:** Comparison with other reported BPFs.

Ref.	Substrate, Process	IL (dB)	RL (dB)	Chip Area	FBW (%)	CFC/TZC	TAR (%)	MTV (MHz)	ATV (MHz)
[7]	Not Given microstrip	2.5–7.72	>15	Not Given (0.095 λ_0_ × 0.058 λ_0_)	10.2–24.3	Yes/No	11/ N.A.	90/ N.A.	125/ N.A.
[8]	RO4003, microstrip	1.4	10	29.4 mm × 29.4 mm Not Given	34.8–56.5	Yes/No	3.6/ N.A.	>30/ N.A.	>142/ N.A.
[9]	RO4003C, microstrip	0.78–2.02	<50	18.73 mm × 11.68 mm Not Given	14.0–40.3	Yes/No	8.3/ N.A.	>5/ N.A.	>100/ N.A.
[10]	RT/Durid6010, Not Given	1.8–4.6	<40	Not Given (0.12 λ_0_ × 0.08 λ_0_)	N.G.	Yes/No	25/ N.A.	>70/ N.A.	>150/ N.A.
[11]	Al_2_O_3_, SIW	2.6–3.3	15–18	12.5 mm × 9.5 mm (0.10 λ_0_ × 0.34 λ_0_)	5.0–9.0	Yes/Yes	33/ 33	>150/ >250	>200/ >300
[12]	Rogers RT 4003, SIW	>2.25	>50	13.0 mm × 13.0 mm Not Given	3.5/6.0	Yes/No	>5/ N.A.	>10/ N.A.	>50/ N.A.
[13]	Rogers 4350, microstrip	<1	>10	18 mm × 18 mm Not Given	8.0	Yes/No	12.5/ N.A.	>400/ N.A.	513/ N.A.
[14]	Rogers 5880, microstrip	0.78–1.18	N.G.	45.0 mm × 45.0 mm Not Given	15.2–16.4	Yes/No	25/ N.A.	54/ N.A.	60/ N.A.
[15]	GaAs, IPD	0.38	17.4	0.800 mm × 0.988 mm (0.015 λ_0_ × 0.018 λ_0_)	51.3	Yes/Yes	20/ 20	103/ 117	128/ 290
[16]	GaAs, IPD	0.62	28.8	1.19 mm ×1.01 mm Not Given	72.7	Yes/Yes	>25/ >25	>100/ >300	>125/ >375
This work	GaAs, IPD	0.38	21.5	0.800 mm × 0.988 mm (0.015 λ_0_ × 0.018 λ_0_)	74.7	Yes/Yes	2.5/ 2.5	1.0/ 1.0	4.7/ 12.8

## Data Availability

Not applicable.

## References

[1] Tsai H., Chen N., Jeng S. (2013). Center frequency and bandwidth controllable microstrip bandpass filter design using loop-shaped dual-mode resonator. IEEE Trans. Microw. Theory Tech..

[2] Aliqab K., Hong J.S. (2019). UWB balanced BPF using a low-cost LCP bonded multilayer PCB technology. IEEE Trans. Microw. Theory Tech..

[3] Lu D., Tang X.H., Barker N.S., Li M., Yan T.F. (2018). Synthesis-applied highly selective tunable dual-mode BPF with element-variable coupling matrix. IEEE Trans. Microw. Theory Tech..

[4] Zheng X., Pan Y., Jiang T. (2018). UWB bandpass filter with dual notched bands using T-shaped resonator and L-shaped defected microstrip structure. Micromachines.

[5] Zhang H., Kang W., Wu W. (2018). Miniaturized dual-band SIW filters using E-shaped slot lines with controllable center frequencies. IEEE Microw. Wirel. Compon. Lett..

[6] Jia T., Ye J., Liu Z. A RF-MEMS based dual-band tunable filter with independently controllable passbands. Proceedings of the 2014 12th IEEE International Conference on Solid-State and Integrated Circuit Technology ICSICT.

[7] Gao L., Zhang X.Y., Zhao X., Zhang Y., Xu J. (2016). Novel compact quad-band bandpass filter with controllable frequencies and bandwidths. IEEE Microw. Wirel. Compon. Lett..

[8] Xu J., Zhu Y. (2017). Tunable bandpass filter using a switched tunable diplexer technique. IEEE Trans. Ind. Electron..

[9] Cheng T., Tam K. (2017). A wideband bandpass flter with reconfigurable bandwidth based on cross-shaped resonator. IEEE Microw. Wirel. Compon. Lett..

[10] Pal B., Mandal M.K., Dwari S. (2019). Varactor tuned dual-band bandpass filter with independently tunable band positions. IEEE Microw. Wirel. Compon. Lett..

[11] Zhu H., Abbosh A.M. (2016). Tunable balanced bandpass filter with wide tuning range of center frequency and bandwidth using compact coupled-line resonator. IEEE Microw. Wirel. Compon. Lett..

[12] Zheng Y., Sazegar M., Maune H., Zhou X., Binder J.R., Jakoby R. (2011). Compact substrate integrated waveguide tunable filter based on ferroelectric ceramics. IEEE Microw. Wirel. Compon. Lett..

[13] Han Y.K., Deng H.W., Zhu J.M., Xing S.B., Han W. (2021). Compact dual-band dual-mode SIW balanced BPF with intrinsic common-mode suppression. IEEE Microw. Wirel. Compon. Lett..

[14] Psychogiou D., Sadasivan K. (2019). Tunable coaxial cavity resonator-based filters using actuated liquid metal posts. IEEE Microw. Wirel. Compon. Lett..

[15] Zhou W., Tang H., Chen J. (2017). Novel microfluidically tunable differential dual-mode patch filter. IEEE Microw. Wirel. Compon. Lett..

[16] Wu Y., Qiang T., Wang C., Adhikari K.K., Lv X., Wu Y. (2019). GaAs-based IPD-fabricated center-frequency-controllable bandpass filter with asymmetrical differential inductor and air-bridge enhanced capacitor. IEEE Access.

[17] Wang Z., Kim E., Liang J., Kim N. (2019). QFN-packaged bandpass filter with intertwined circular spiral inductor and integrated center-located capacitors using integrated passive device technology. IEEE Access.

[18] Zhou H., Götzinger M., Peukert W. (2003). The influence of particle charge and roughness on particle–substrate adhesion. Powder Technol..

[19] Nilsson J., Borg J., Johansson J. (2019). Maximal Q factor for an on-chip, fuse-based trimmable capacitor. Electronics.

[20] Bahl I.J. (2003). Lumped Elements for RF and Microwave Circuits.

[21] Qiang T., Wang C., Kim N. (2016). A compact high-reliability high-performance 900-MHz WPD using GaAs-IPD technology. IEEE Microw. Wirel. Compon. Lett..

[22] Mohan S.S., Hershenson M.D., Boyd M.S.P., Lee T.H. (1999). Simple accurate expressions for planar spiral inductances. IEEE J. Solid St. Circ..

[23] Choi B.H., Jin Z.J., Kim M.G., Sun H., Yun T.Y. (2008). Equivalent-circuit model based on mathematical analysis for multilayer chip inductors. IET Microw. Antenna P.

[24] Haobijam G., Palathinkal R.P. (2013). Design and Analysis of Spiral Inductors.

[25] Chen J. (2006). On-Chip Spiral Inductor Transformer Design and Modeling for RF Applications. Ph.D. Thesis.

[26] Yue C.P., Wong S.S. (2000). Physical modeling of spiral inductors on silicon. IEEE Trans. Electron. Devices.

[27] Wang Z.J., Kim E.S., Liang J.G., Qiang T., Kim N.Y. (2018). A high-frequency-compatible miniaturized bandpass filter with air-bridge structures using GaAs-based integrated passive device technology. Micromachines.

